# Short review: novel concepts in the approach to patients with amiodarone-induced thyrotoxicosis

**DOI:** 10.1007/s40618-023-02168-3

**Published:** 2023-09-20

**Authors:** D. Cappellani, L. Bartalena, F. Bogazzi

**Affiliations:** 1 Department of Clinical and Experimental Medicine, Unit of Endocrinology, University of Pisa, Ospedale Cisanello, via Paradisa 2, 56124 Pisa, Italy; 2 School of Medicine, University of Insubria, Varese, Italy

**Keywords:** Amiodarone, Thyrotoxicosis, Hyperthyroidism, Cardiovascular, Thyroidectomy

## Abstract

**Introduction:**

Amiodarone-induced thyrotoxicosis is associated with high morbidity and mortality rates. The approach to this condition is widely variable across different medical specialists and even among expert endocrinologists. As a matter of fact, the approach to amiodarone-induced thyrotoxicosis has always been considered difficult, due to diagnostic uncertainties easily resulting in missteps, and therapeutic challenges easily resulting in unresponsiveness or slow-responsiveness to the administered drugs.

**Purpose:**

Our purpose is to review novelties emerged during the last years about this condition, with the aim to provide novel insights on the diagnostic and therapeutic management of this challenging condition.

## Introduction

In about 15–20% of patients, amiodarone therapy results in thyroid dysfunction, namely amiodarone-induced hypothyroidism or amiodarone-induced thyrotoxicosis (AIT) [1]. There are two main different AIT types: type 1 amiodarone-induced thyrotoxicosis (AIT1) is a form of iodine-induced hyperthyroidism, which develops when a thyroid with subtle functional autonomy is exposed to iodine excess contained in the amiodarone molecule; type 2 amiodarone-induced thyrotoxicosis (AIT2) is a destructive thyroiditis, which may develop even in a normal thyroid gland and is due to the leakage of preformed thyroid hormones from damaged thyroid follicles [2]. Indefinite or mixed forms also exist [1].

Although less common than amiodarone-induced hypothyroidism [3, 4], AIT is a worrisome condition, due to the following:

(i) difficulties in the differential diagnosis between the two main AIT types, which is fundamental for a correct therapeutic approach;
(ii) frequent lack of a prompt response to medical therapies, often resulting in uncertainties about appropriateness of the initial therapeutic approach;
(iii) underlying heart disease, which may deteriorate with increasing time of exposure to thyroid hormone excess, thereby resulting in high morbidity and mortality rates.

For all the above reasons, it is not surprising that surveys carried out among expert endocrinologists from different geographical areas unveiled important ambiguities in AIT diagnosis and treatment [5–7]. Likewise, a recent large real-life study demonstrated significant differences in the approach to AIT among different medical specialties with high percentages of physicians (up to 65% of the total) administering first line-therapies outside the current standard of care [8]. In 2016, the American Thyroid Association published the guidelines for the management of hyperthyroidism/thyrotoxicosis, which included a section dedicated to the management of AIT [9]. Two years later, the European Thyroid Association published a guideline specifically dedicated to amiodarone-induced thyroid dysfunction [10]. Beside a few differences between the two guidelines, the analysis and description of which is beyond the purposes of the present review, herein we want to summarize novelties emerging from the latest studies published after the guidelines, which may introduce some changes in the approach to the management of this complex disease.

## Novel concepts emerging in AIT diagnosis

Several diagnostic procedures are often required for a clear-cut differentiation of the two AIT types, and differential diagnosis is crucial, since the two types of AIT warrant different treatments. The tools for the differential diagnosis of the AIT types have been refined over the years: the oldest papers considered AIT1 as a disease developing on an underlying and preexisting thyroid disease (nodular or autoimmune), and AIT2 as a disease developing on a previously normal thyroid gland [11–13]. Although this holds true in the majority of cases, it is now clear that the presence of an underlying and preexisting thyroid disease does not necessarily exclude a diagnosis of AIT2 [14–16]. Tools available for the differential diagnosis of the two AIT main types are summarized in Table 1.

**Table 1:** Diagnostic approach to amiodarone-induced thyrotoxicosis

	AIT1	AIT2
Anamnesis		
Preexisting thyroid disease	Yes	Usually no
Onset time	Usually short (median 3 months)	Usually long (median 30 months)
Lab		
Thyroid hormone concentrations	Mean value usually higher in AIT2*	
FT4/FT3 ratio	May be higher (> 4) in AIT2*	
Anti-thyroglobulin and anti-thyroperoxidase autoantibodies	Present if Graves’ disease is the underlying thyroid abnormality	May be present
Thyroid receptor antibodies (TSHR-Ab)	Present with stimulatory activity if Graves’ disease is the underlying thyroid abnormality	May be present without stimulatory activity
Serum cell damage markers (i.e., IL6 and PCR)	Useless	
Imaging		
Thyroid ultrasound	Thyroid nodules frequently present (if nodular goiter is the underlying thyroid abnormality)	Thyroid nodules may be present
Color-flow Doppler sonography	Increased vascularization	Absent hypervascularization
Radioactive iodine uptake (131-I)	Low/normal/increased uptake	Absent/low uptake
Thyroid scintigraphy with cell damage markers (i.e., 99mTc-sestaMIBI)	More studies required	
Unenhanced CT scan	More studies required	

*AIT1* type 1 amiodarone-induced thyrotoxicosis, *AIT2* type 2 amiodarone-induced thyrotoxicosis, *TSHR-Abs* TSH-receptor antibodies

*may be not useful in the single patient

Thyroid hormones concentrations are not useful to differentiate type 1 and type 2 AIT, although they tend to be higher in type 2 AIT [15, 17]. In the past, an increased T4/T3 ratio (> 4) was considered to be a feature of type 2 AIT, as derived from populations studies [17, 18], but this parameter is not useful in the individual patient due to the large overlap in the ratios between the two AIT types [15, 17]. However, when present, it might be considered.

Type 2 AIT has been historically considered not to be associated with signs of autoimmunity [5, 19]. However, the presence of anti-thyroglobulin and/or anti-thyroperoxidase autoantibodies cannot rule out a diagnosis of AIT2: this concept, which has been already included in the most recently published guidelines [10], was derived from a study demonstrating that a significant proportion of patients receiving a diagnosis of AIT2 had detectable thyroid autoantibodies, and this had no implications on the response to pharmacologic therapy with glucocorticoids [20]; the same study showed that the presence of thyroid autoantibodies was associated with a significantly higher rate of hypothyroidism following glucocorticoid therapy for AIT2 (26% vs 5% in thyroid autoantibody-negative patients; *p = *0.032).

More recently, the role of thyrotropin receptor autoantibodies (TSHR-Ab) in the differential diagnosis between the two AIT types has been questioned: as a matter of fact, the presence of TSHR-Ab was found in patients classified as either AIT1 or AIT2 on the basis of clinical and histopathological features [21]. A functional assay demonstrated that only TSHR-Ab obtained from patients displaying AIT1 and Graves’ disease features stimulated the TSH receptor, whereas TSHR-Ab obtained from AIT2 patients displayed a neutral or inhibiting activity [21]. The presence of TSHR-Ab is reported in the most recent guidelines as a distinctive marker of AIT1 in the form of amiodarone-induced Graves’ disease [10], but this point probably needs to be revised in future guidelines. TSHR-Ab assays measuring inhibition of binding to the TSH receptor (so-called TBII assays, i.e., thyrotropin-binding inhibitor immunoglobulin assays) cannot establish whether TSHR-Ab exert a stimulatory, neutral, or inhibitory effect [22]. At variance, assays measuring the stimulatory activity of the TSHR-Ab on the TSH receptor (so-called TSI assays, i.e., thyroid stimulating immunoglobulin assays) [23, 24] do not measure TSHR-Ab with a neutral or inhibitory effect. Even though the use of TSI assays has not been reported to date in the setting of AIT, it may be anticipated that the use of TSI in the clinical practice may help to reduce misclassification of TSHR-Ab-positive AIT patients [18].

Thyroid ultrasonography is useful for the assessment of thyroid volume, presence/absence of nodules, parenchymal echogenicity, and vascularization [10]. Even though it is not uncommon that silent areas of functional autonomy are present within a nodular goiter, conventional echography alone does not provide functional information, and the presence of nodules or goiter does not necessarily imply a diagnosis of type 1 AIT [15]. Color-flow Doppler sonography shows increased thyroid vascularization in most patients affected by type 1 AIT and absent hypervascularization (in spite of high serum thyroid hormone concentrations) in patients with type 2 AIT [10, 25], making this procedure an important tool to differentiate AIT types.

The evidence of increased thyroid density at unenhanced CT scan of AIT2 patients was originally reported in 2018 [26]. In details, the authors reported the incidental finding in an AIT2 patient of a “white thyroid” resembling a thyroid image on an enhanced CT scan; a subsequent comparison between thyroid densities (measured in Hounsfield units) of AIT2 patients compared to either patients with no thyroid dysfunction under amiodarone treatment or to healthy controls revealed a significant decrease when moving from the former to the latter [26]. This study was further supported by a report of a single patient who underwent several CT scans before and along the course of AIT2: as a matter of fact, thyroid density increased after amiodarone administration and before detection of AIT, peaked after initiation of treatment for thyrotoxicosis, and returned to normal when thyrotoxicosis remitted [27]. Being the observation of the “white thyroid” subsequent to the publication of the most recent guidelines [9, 10], this diagnostic tool is currently not included in the AIT workup. Given the small number of patients studied with this technique, the application of unenhanced CT scan in the diagnostic armamentarium for AIT remains unclear, and larger population studies are warranted.

Thyroidal radioactive iodine uptake (RAIU) has a diagnostic value, since most patients with type 1 AIT present low-to-normal RAIU values, whereas those with type 2 have low-to-undetectable values [10, 12, 13]; this information is derived from historical studies mainly performed in Italy, which was a iodine-deficient area at that time, and its applicability in iodine sufficient areas has been questioned [28]. However, at least in Italy, thyroidal RAIU remains a useful tool for differentiating the two main AIT types. During the course of the years, different studies assayed the possible application of radionuclides as markers of tissue destruction: the first study on 99mTc-sestaMIBI (the uptake of which is expected to be reduced in destructive AIT2 type) as a tool for the differential diagnosis between the two main AIT types was published in 2008 [29]. Two subsequent studies, recognizing that the information to guide reporting with this diagnostic technique is qualitative and highly subjective, proposed the use of a target-to-background ratio, with the aim to reduce the interobserver variability and, thus, improve the reliability of this method [30, 31]. The major criticism to this technique remains the low sensitivity for AIT1 and the low specificity for AIT2, as detailed in the 2018 ETA Guidelines [10]. However, during the most recent years, scattered reports of the use of 99mTc-sestaMIBI in the AIT workup have been published [32–34], including a small functional imaging-histopathologic correlation study [35]. Thus, even though 99mTc-sestaMIBI does not currently seem to have enough reliability to be included in the recommended workup, it remains an interesting research topic.

Finally, an important emerging point is that a scrupulous and thorough differential diagnosis between the AIT types may be not mandatory in case of underlying severe/precipitating cardiac dysfunction. As a matter of fact, such situations may warrant a prompt surgical management (i.e., salvage thyroidectomy) to be performed urgently before restoration of euthyroidism by medical therapies [10, 36]. This issue will be discussed in details in the following section.

## Novel concepts emerging in AIT therapy

The “traditional” approach to AIT was centered on the control of thyrotoxicosis. Original studies were thus focused on the medical approach to AIT with the restoration of euthyroidism as the primary outcome [37–41]. The paper published in 2007 by Conen and colleagues considered, for the first time, the event-free survival as their primary specific outcome, highlighting that 56% of patients reached this end-point [42]. In this study, medical therapies were compared with regard to the incidence of cardiovascular events and not only with regard to control of thyrotoxicosis. Moreover, the importance of left-ventricular ejection fraction (LVEF) as a prognostic marker for AIT patients was underscored, by demonstrating that impaired LVEF was associated with an increased cardiovascular events rate both during the course of AIT and after restoration of euthyroidism [42]. The high rate of cardiovascular events and mortality consequent to AIT was thereafter confirmed in a large population of amiodarone-treated patients [43]. Therefore, the most recent literature regarding AIT was focused not only on the thyroid (i.e., control of thyrotoxicosis), but also on the heart, (i.e., development of cardiovascular complications and mortality) [8, 44].

Medical therapy for AIT1 is represented by thionamides administered at high dosage for a prolonged period of time: the ATA guidelines suggest a starting dose of methimazole 40 mg per day (or equivalent of propylthiouracil) [9], whereas the ETA guidelines suggest a starting dose of methimazole 40–60 mg per day (or equivalent of propylthiouracil) [10]. The add-on of perchlorate, which was originally proposed with the aim to reduce iodine uptake, thus increasing thyroidal sensitivity to thionamides [45], is considered only by the ETA guidelines: however, due to concerns regarding possible adverse events associated with this medication, it is recommended not to exceed 1 g per day of perchlorate and not to prolong treatment for more than 4–6 weeks [10].

Medical therapy for AIT2 is based on glucocorticoids, and thionamides [40] or perchlorate [41] have no role in this AIT type. The ATA Guidelines recommend starting therapy with prednisone 40 mg per day (or equivalents) [9], whereas the ETA Guidelines recommend starting therapy with prednisone 30 mg per day (or equivalents) [10]. A long course of glucocorticoid therapy may be required, with baseline thyroid hormone concentrations and thyroid volume being significant predictors of the time required to restore euthyroidism in this setting [46]. Currently, no head-to-head randomized clinical trial comparing different glucocorticoid treatment schedules has been published.

A recent study demonstrated that in both AIT1 and AIT2 administration of the appropriate medication (i.e., thionamides for AIT1 and glucocorticoids for AIT2) but at a lower dosage than recommended by the guidelines was equivalent to administering no therapy at all in terms of control of thyrotoxicosis and development of cardiovascular complications [8].

For those AIT patients in whom a clear differential diagnosis of AIT type cannot be made (i.e., indefinite AIT type), or for those for whom a contribution of both iodine-induced hyperthyroidism and destructive thyroiditis (i.e., mixed AIT type) is suspected, guidelines recommend considering a combination of thionamides and glucocorticoids. In particular, ATA guidelines suggest this approach whenever in doubt [9], whereas ETA guidelines suggest considering whether to start immediately a combination therapy or to start a trial of thionamides for 4–6 weeks and add glucocorticoids only when response is poor [10].

Thionamides’ administration has been recently associated with a higher risk of adverse events in AIT patients compared to other hyperthyroid patients: a large registry study performed in Israel identified a higher rate of thionamides-induced bone marrow adverse events in AIT patients compared to other causes of hyperthyroidism, including Graves’ disease (1.35 vs 0.14% *p < *0.0001) [47]. Furthermore, another real-life study demonstrated that combination therapy does not provide any advantage in terms of control of thyrotoxicosis or development of cardiovascular complications and hospitalizations compared to the optimal medical therapy for the specific AIT type [8]. In the same context, combination therapies were confirmed as associated with higher rates of adverse events, mostly related to thionamides, and subsequent hospitalizations. Therefore, when the diagnosis of a mixed AIT type is substantiated, combination therapy is justified; conversely, in the case of indefinite AIT type, all the possible diagnostic efforts should be made before deciding upon a combination therapy [8].

Total thyroidectomy was originally proposed as a therapeutic option for AIT in 1990 [48]. Many reports and studies regarding total thyroidectomy for the management of AIT were published during the last decade. An original series reported very high rates of complications (29.5%) and mortality (8.8%)[49], even though a more recent study on 17 AIT patients submitted to total thyroidectomy at the same hospital revised the unfavorable data previously described [49]. Importantly, subsequent reports were generally concordant in confirming the safety of surgery in this setting, when performed by a skilled team of anesthesiologists and surgeons [17, 50–53]: specifically, the two largest series (totalling 102 thyroidectomies for AIT) reported complications rate ranging from 7.8% to 11.8% [17, 54].

In a large population study comparing total thyroidectomy to optimal medical therapies for AIT, as recommended by the guidelines a significantly low operative mortality (1.9%) was reported [17]. A longer follow-up showed that for AIT patients affected by moderate-to-severe left-ventricular systolic dysfunction (i.e., LVEF < 40%, as defined by the Framingham Heart Study [55]), surgery presented a significant survival advantage compared to optimal medical therapies, both when considering cardiovascular mortality at 5 years and overall mortality at 10 years [17]. The same survival advantage was not confirmed for patients affected by less severe cardiac dysfunction [18]. The favorable result obtained when surgery was performed in this high-risk setting was ascribed to its capability to promptly restore euthyroidism with a consequent improvement in systolic function (mean LVEF 45% pre-surgery vs 50% post-surgery, *p < *0.0001) [17]. This has been confirmed by other studies, particularly in patients with severe systolic dysfunction [54, 56].

An important issue is the timing of total thyroidectomy, whether surgery should be performed promptly while the patient is thyrotoxic or later after preliminary restoration of euthyroidism by pharmacological therapies. A relatively small cohort study of 11 AIT patients no significant intra- and postoperative complications when thyroidectomy was performed in the thyrotoxic state or electively after restoration of euthyroidism [50]. This issue was expanded in a subsequent study where early and delayed thyroidectomies were compared not only in terms of surgical complications, but also in terms of peritreatment and 5-year cardiovascular mortality [57]. Patients were clustered according to severity of the underlying cardiac disease and compared according to the promptness of surgery, urgent surgery while thyrotoxic *versus* elective surgery after restoration of euthyroidism [57]. Patients affected by moderate-to-severe left-ventricular systolic dysfunction benefited from early thyroidectomy while thyrotoxic: in this setting, peritreatment mortality rate differed significantly according to the promptness of surgery (0% for those submitted while thyrotoxic vs 40% for those submitted to surgery after restoration of euthyroidism), with important implications on the 5-year overall cardiovascular mortality rates (12 vs 53%, respectively) [57]. At multivariate analysis, duration of exposure to thyroid hormone excess was confirmed as a significant risk factor for mortality (HR 1.004, IQR 1.000–1.006) [57], as previously postulated by other authors [58]. The same results were recently confirmed in another study from a different country which reported a baseline LVEF < 40% and a longer time period prior to surgery as the main determinants of cardiac mortality [54].

It is important to highlight that timely application of the appropriate pharmacological therapy for AIT seems to reduce both hospitalization rates and the need for urgent thyroidectomies [8]. Whether this point has an impact on the development of surgery-related adverse events remains to be investigated.

Total thyroidectomy seems to represent a valuable therapeutic option in limited and well-defined settings, in line with the guidelines’ recommendations [9, 10]. Due to its capability to promptly restore euthyroidism, salvage thyroidectomy can be considered the treatment of choice for those patients presenting with a thyrotoxicosis-driven rapid deterioration of cardiac function and/or malignant arrhythmias. As anesthetic and surgical risks may remain quite high in this setting [59], a careful risk-to-benefit evaluation should be performed in the individual patient [60, 61]. It seems agreeable to have surgery performed by skilled surgeons and only when a multidisciplinary team is available [17, 53, 54, 62, 63].

High-dose intravenous glucocorticoids were proposed as an alternative to total thyroidectomy for AIT2 patients affected by severe thyrotoxicosis and unresponsive to standard oral therapies [64]. The original proposed scheme encompassed the administration of intravenous methylprednisolone twice per week for a limited time and inter-pulse daily oral glucocorticoids (5–11 weeks), followed by other 3–11 weeks of oral glucocorticoids alone, resulting in a prompt restoration of euthyroidism without significant side-effects. This favorable results were not confirmed in another exploratory case–control study, where intravenous glucocorticoids were administered without oral inter-pulse glucocorticoids, and were deemed responsible for reducing free thyroid hormone concentrations in a fashion similar to standard-of-care oral glucocorticoids while exposing patients to an overall higher glucocorticoid dosage [65]. These conflicting results come from two small studies differing both for patients’ selection and study protocol, thus requiring further studies performed on larger populations. Currently, due to paucity of data and conflicting results, high-dose intravenous glucocorticoids cannot be recommended as salvage therapy in compromised patients.

**Fig. 1:**
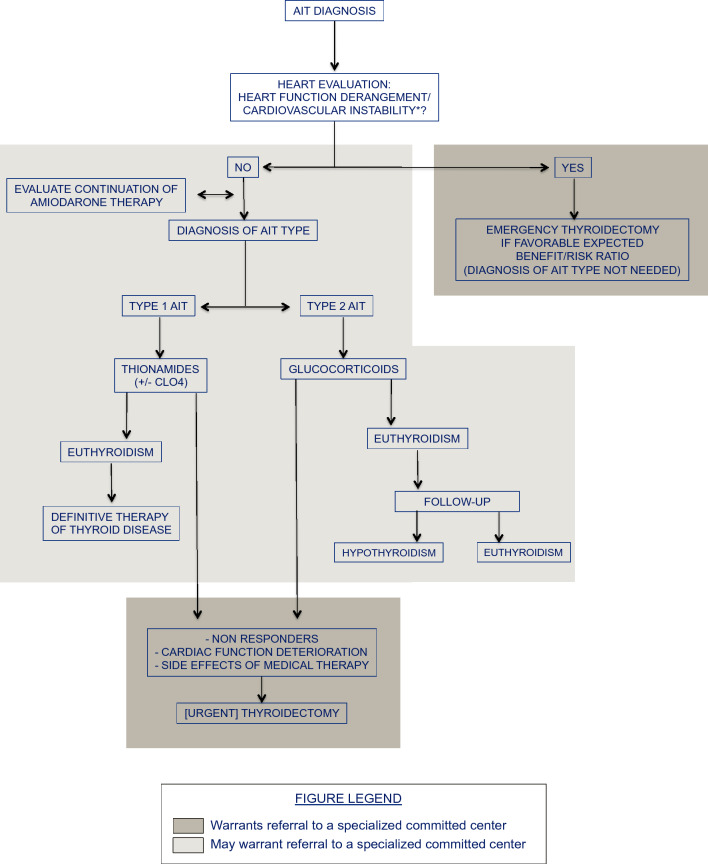
Treatment approach to amiodarone-induced thyrotoxicosis: a flowchart. *AIT* amiodarone-induced thyrotoxicosis. *ClO4-* perchlorate. *Heart function derangement/cardiovascular instability: i.e., presence or high risk of life-threatening severe congestive heart failure and/or malignant arrhythmias

A flowchart of AIT management according to above-mentioned novel concepts is reported in Fig. 1.

## Conclusions

A contemporary approach to patients with AIT should take into account the following features as prognostic outcomes:

- underlying cardiac disease, sometimes in a precarious balance;
- thyrotoxicosis, often severe;
- long-lasting thyroid disease, due both to frequent diagnostic and therapeutic delays and to initial unresponsiveness to standard therapies.

During the last years, the approach to AIT shifted from considering therapies solely in terms of control of thyrotoxicosis (i.e., endocrine perspective), to considering therapies in terms of prevention of cardiovascular events, hospitalizations, and mortality (i.e., general perspective).

Accordingly, the patient-centered approach to AIT should be tailored on the following premises:

1. Consideration of the underlying cardiac conditions: in case of severe/rapidly deteriorating and life-threatening cardiac conditions, patient should undergo emergency total thyroidectomy without the need of a differential diagnosis between AIT types.
2. Careful AIT type diagnosis: if an emergency treatment is not needed, an exhaustive, albeit prompt, diagnostic approach should be made, to identify the AIT type and select the appropriate pharmacologic approach.
3. Reconsideration of the need of an urgent thyroidectomy at any time during the course of the disease in case of deterioration of the cardiac conditions.

## Data Availability

Data sharing is not applicable to this article as no datasets were generated or analysed during the current study.
